# A Systematic Review on the Implication of Minerals in the Onset, Severity and Treatment of Periodontal Disease

**DOI:** 10.3390/molecules21091183

**Published:** 2016-09-07

**Authors:** Alfonso Varela-López, Francesca Giampieri, Pedro Bullón, Maurizio Battino, José L. Quiles

**Affiliations:** 1 Department of Physiology, Institute of Nutrition and Food Technology “Jose Mataix”, University of Granada, Biomedical Research Center, Avda. Conocimiento s.n., 18100 Armilla, Granada, Spain; alvarela@ugr.es; 2 Dipartimento di Scienze Cliniche Specialistiche ed Odontostomatologiche (DISCO)-Sez. Biochimica, Facoltà di Medicina, Università Politecnica delle Marche, 60131 Ancona, Italy; f.giampieri@univpm.it (F.G.); m.a.battino@univpm.it (M.B.); 3 Department of Stomalogy, Dental School, University of Sevilla, C/Avicena s.n., 41009 Sevilla, Andalusia, Spain; pbullon@us.es

**Keywords:** mineral, calcium, periodontitis, oral health, diet, nutrition

## Abstract

Periodontal disease is an inflammatory disease with high prevalence in adults that leads to destruction of the teeth-supporting tissues. Periodontal therapy has been traditionally directed at reduction of the bacterial load to a level that encourages health-promoting bacteria and maintenance of oral-hygiene. The role of nutrition in different chronic inflammatory diseases has been the subject of an increasing body of research in the last decades. In this sense, there has been an important increase in the volume of research on role of nutrition in periodontitis since the diet has known effects on the immune system and inflammatory cascades. Minerals play a key role in all these processes due to the multiple pathways where they participate. To clarify the role of the different minerals in the establishment, progression and/or treatment of this pathology, a systemically review of published literature cited in PubMed until May 2016 was conducted, which included research on the relationship of these elements with the onset and progression of periodontal disease. Among all the minerals, calcium dietary intake seems important to maintain alveolar bone. Likewise, dietary proportions of minerals that may influence its metabolism also can be relevant. Lastly, some observations suggest that all those minerals with roles in immune and/or antioxidant systems should be considered in future research.

## 1. Introduction

Periodontal disease is an inflammatory disease that leads to destruction of the teeth-supporting tissues (i.e., the periodontium). The term mainly includes gingivitis, chronic periodontitis and aggressive periodontitis [1]. Periodontitis is a ubiquitous disease with high prevalence in adults. In particular, the WHO sponsored Global Burden of Diseases study indicates that 11.2% of adults worldwide experience severe periodontal disease. This disease is considered the result of the interaction between pathogenic bacteria and the host’s immune response [2,3]. Dental plaque biofilm that is formed by bacteria and toxins is the primary etiology [4,5]. Periodontal disease is initiated by the accumulation of a pathogenic biofilm at and below the gingival margin [6]. In turn, bacterial product or components stimulate polymorphonuclear and other resident cells which may damage connective tissue surrounding the teeth if their stimulation is excessive [4,5,7]. As consequence, a progressive breakdown of the periodontal ligament and alveolar bone accompanied by periodontal pocket formation, gingival recession or both occur [8]. It is considered that both local and systemic factors affect the severity and progression of periodontal disease. There are certain groups of “dysbiotic” microorganisms (“pathogenic species”) considered as causative agents [9] whose amount would increase against microorganisms that co-exist symbiotically with a proportionate host response that is in turn associated with resolution of destructive inflammatory cascades. These usually are some Gram-negative bacteria species that drive excess inflammation in periodontitis susceptible subjects that would have a “hyper-inflammatory” response [6]. Thus, it is thought that host response and immune system are the actual determinants of this disease, at least in many cases [9].

Periodontal therapy has been traditionally been directed at reduction of the bacterial load to a level that encourages health-promoting bacteria and maintenance of oral-hygiene [6,10]. However, because of systemic factors also seem to affect periodontal disease, other research topics have appeared in last decades. The role of nutrition in different chronic inflammatory diseases has been the subject of an increasing body of research in the last decades. In this sense, there has been an important surge in the amount of research on the role of nutrition in periodontitis since the diet has known effects on the immune system and inflammatory cascades. Understanding the role and implication of different nutrients in this disease should allow identifying nutritional risk factors and modulators of periodontal inflammation for targeted prevention and treatment approaches in patients with specific nutritional depletion. This is particularly interesting for people with factors that comprise the ability to absorb or metabolize key dietary nutrients due to different causes, including genetics [11].

Nutrients can be categorized as either macronutrients (needed in relatively large amounts and in most of cases to provide energy) or micronutrients. These last are dietary compounds that do not provide energy but are required by living organisms and are essential for optimal health, proper growth, and metabolism. It has been suggested that periodontitis is associated with deficiencies in serum micronutrient levels that usually increase the risk of disease [12]. These basically include vitamins and minerals [12]. Minerals make up about 4% of the body weight and they are mainly located in the skeleton, enzymes, hormones and vitamins. They usually act as cofactors in enzymes and their presence in balanced concentrations in different body compartments is needed for multiple physiological processes (heart rhythm, muscle contraction, nerve conduction, and acid-base balance homeostasis). Moreover they also have structural functions that are particularly important for bones and teeth. Minerals can be classified as either major minerals (>100 mg/day) or trace elements (<100 mg/day). The major minerals are sodium, potassium, calcium, magnesium, phosphorus and sulfur. The trace minerals are: iron, zinc, iodine, selenium, fluoride, copper, cobalt, chromium, manganese and molybdenum [1,13].

Numerous reviews about the association between periodontitis and different components of the diet have been published [1,6,9,10,11,12,14,15,16,17,18], but only a few of them used a systematic approach [9,11,12,19]. Moreover, they were restricted to certain population groups [11], study types [9,11,12] or did not include animals [1,6,9,11,12,14,15]. This paper systemically reviews the literature available in the PubMed database the inception of the database until May 2016 on the effects of different mineral intakes or nutritional status with the onset, severity and treatment of periodontal disease both in humans and animals, attending with special interest to dietary interventions and the implications of each one for the mechanisms involved in these pathologies.

## 2. Results

**Figure 1:**
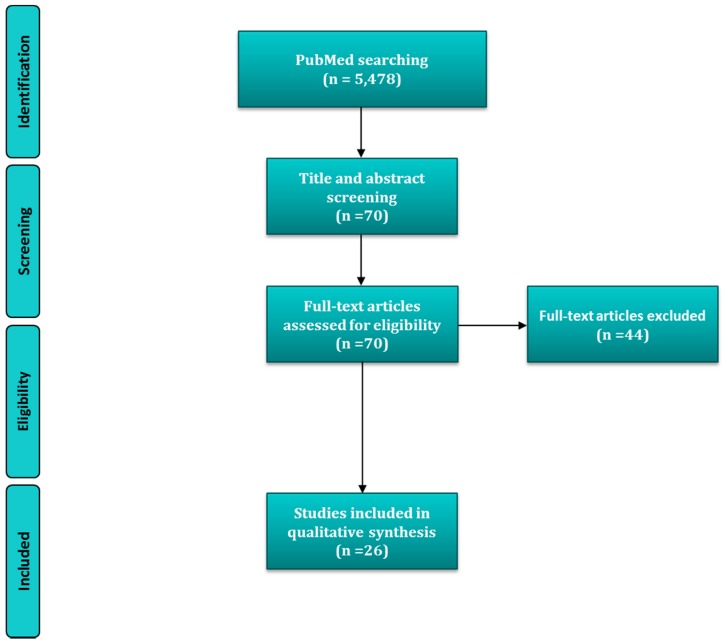
Flow diagram and search strategy.

The search terms used provided a total of 5478 items. After abstract screening this number was reduced to 70 potential articles to be included. Finally, a comprehensive full-text reading was performed and 44 more were discarded (Figure 1). Overall, there were data for nine of the different minerals: calcium, magnesium, phosphorus, iron, potassium, copper, zinc, selenium and manganese, which are presented in their respective subsections. Some of the included studies investigated potential associations or effects for two or more different minerals and consequently, they were repeated in different subsections.

### 2.1. Calcium

With the methodology used, 15 publications about the role of calcium in periodontal disease were selected, specifically 12 observational studies in humans [20,21,22,23,24,25,26,27,28,29] and five experimental studies in animals [30,31,32,33,34]. Dietary intake estimates were obtained in nine of them [20,21,22,23,24,25,26,28,29]. Most of them were cross-sectional studies, but there are also a case-control [28] and a cohort study [29] (Table 1). In addition, three of them also included serum calcium levels [20,22]. Likewise, there were a cross-sectional study [35] and a case-control study [27] where serum calcium level was the sole variable related to this nutrient analyzed. Anyway, it is important to point out that serum calcium levels are affected by nutritional deficiencies only in severe cases, because of homeostasis processes that control it. However, possible associations between calcemia and periodontal health could help us to understand the importance of this mineral. Moreover the use of calcium-antagonist drugs [36] was also evaluated in a descriptive investigation. In addition to these studies, other two discussed calcium to magnesium ratio in blood [36,37], but they will be covered below together with other investigations about magnesium.

Regarding cross-sectional studies, various clinical outcomes have been used to evaluate periodontal disease or periodontitis severity. In one of the cross-sectional studies that utilized data from 11,787 subjects who participated in the National and Health Nutrition Examination Survey (NHANES) III, Nishida et al. [20] found an inverse association among dietary intakes and prevalence of periodontal disease in younger subjects (20–39 years) with *odds* *ratios* (ORs) of 1.84 (95%CI: 1.36–2.48, *p* < 0.001) for men, and 1.99 (95%CI: 1.34–2.97. *p* < 0.01) for women; and in medium-age men (40–59 years) with an OR of 1.90 (95%CI: 1.41–2.55, *p* < 0.001). However, it was observed that low total serum levels (adjusted by calcium intake) were related to periodontal disease only in younger females with an OR of 6.11 (95%CI: 2.36–15.84, *p* < 0.001). Interestingly, this group of subjects was the only one that revealed a dose-response relationship with a 54% higher risk of periodontal disease for the lowest intake (2–499 mg) and 27% for the moderate intake group (500–799 mg) after adjustment for gingival bleeding and tobacco consumption [20]. Other cross-sectional study was carried out in 3287 adult participants in the 2007–2008 Danish Health Examination Survey (DANHES) who volunteered to participate in an oral health examination. Previously, as part of the DANHES, subjects had filled an Internet-based food frequency questionnaire (FFQ) over a year that was used to estimate dietary intakes of calcium along with other nutrients. Oral examination was used to identify subjects with chronic severe periodontitis [21] defined according to the Centers for Disease Control and Prevention (CDC) and American Academy of Periodontology criteria (AAP) [38]. Intakes of calcium within recommendations (1000 mg/day for women aged under 50 years old and men under 70 years old; or 1200 mg/day women aged 50 years old or more and men aged 70 years old or more) was inversely associated with lower likelihood of severe periodontitis (OR = 0.76, 95%CI: 0.58, 0.99, *p* = 0.041) after adjustment for typical covariates (age, gender, education, smoking, sucrose intake, alcohol consumption, number of teeth, daily brushing, regular visits to the dentist and chronic illness). In the same sense, Freeland et al. [22] showed a correlation coefficient of −0.24 between calcium intake and Russell’s periodontal index (RPI) in a sample of 80 subjects from a dental clinic, although the authors considered a significance level of 0.075. Further, dietary intake did not show any significant association. Finally, other cross-sectional study was focused on pregnant women (*n* = 1162) as a risk group. For this dietary intake data were collected during pregnancy by a diet history questionnaire and post-partum oral examinations were performed. Multiple regression analysis adjusted for typical covariates (age, region of residence, smoking status, toothbrushing frequency, use of an interdental brush, household income, and education) indicated that subjects in the highest quartile of calcium intake was associated with a lower prevalence of periodontal disease than those in the lowest, with a OR of 0.53 (95%CI: 0.30–0.94, *p* = 0.07 for trend) [23].

In turn, for the rest of descriptive papers, no significant relationship with parameters related to periodontal disease was observed. In a study performed among Japanese non-smokers [24], no relationship between calcium intake and percentage of sites with bleeding was found on probing (BOP) or Community Periodontal Index (CPI), although correlations were observed at a bivariate level. Similarly, no differences were found in serum levels were measured in a case-control study comparing chronic and aggressive periodontitis patients defined according to the International Workshop for the Classification of Periodontal Diseases and Conditions in 1999 (IWCPDC) criteria [39] with health individuals from a health center in China. Finally, in a study with adult individuals from the Study of Health in Pomerania (SHIP) survey (Germany) who used blocking calcium-channels drugs, higher periodontal pocket probing depth (PPD), but not clinical attachment loss (CAL), BOP, number of teeth or plaque was found [36].

On the other hand, there was a cross-sectional survey focused on the intake of dairy products with special interest paid to milk, cheese and fermented foods. Among these, a study was conducted only in older adults, but calcium intakes were estimated too [25]. In this, intakes of total dairy calcium (incidence rate ratio [IRR] = 0.97, 95%CI: 0.96–0.99, *p* = 0.021), calcium from milk (IRR = 0.97, 95%CI: 0.95–0.99, *p* = 0.025) and from fermented foods (IRR = 0.96 95%CI: 0.92–0.99, *p* = 0.03), but non-dairy calcium were inversely associated with the severity of periodontitis (determined through Community Periodontal Treatment Needed Index (CPITN) score) after adjustment for several risk factors. Two multivariate analyses indicated differences in calcium intake in the case-control that compared gingivitis-affected and non-affected female adolescents whereas in another it was substituted by riboflavin as we have said above, but it was due to the fact both were intercorrelated with milk consumption [28].

Similar differences existed among cohort studies. In one of the cohort studies that included 2113 medium-age (30–60 years) participants in Danish Monitoring Trends and Determinants in Cardiovascular Disease (MONICA) study (Denmark), it was noted negative associations of calcium intake with number of tooth at baseline and subsequent tooth loss. If dietary recommendations were taken into account, calcium intake bellow recommendations was associated with increased risk of subsequent tooth loss after 6 years, only in men (IRR = 1.70, 95%CI: 1.15–2.48, *p* < 0.05) [26]. On the other hand, it was reported that high calcemia values seem to prevent progression of periodontal disease after 6 years in 70 years old subjects from Niigata city [29].

Animal research was represented by three articles (Table 2), two performed in rats (*Rattus norvegicu*s) [30,31] and one in mice (*Mus musculus*) [34]. Most of them analyzed the effect of dietary calcium deficiencies on alveolar bone [30,31], namely three considering interactions with pregnancy and lactation [30,31]. For this aim, in one of them, female rats were housed with male rats fed diet-containing calcium 0.9%, 0.3%, or 0.02% and after 25 days were subdivided into pregnant and non-pregnant animals. Additionally, pups were maintaining with their mother for lactation [31]. BMD and alveolar bone decreased according to dietary calcium levels in both, adults (*p* < 0.0001) and pups (22.5% less in 0.02% calcium and 12.7% in 0.3% calcium respect to 0.9% calcium diet, *p* < 0.01), but the magnitude was greater in the pregnant group than in non-pregnant when they were fed diets containing calcium 0.02% and 0.3% (*p* < 0.05) [31].

The experimental design in the other study was similar, except that periodontitis was experimentally induced by an elastic ring in the right side of mandible on day 32. Similarly, BMD on diseased sites decreased according to the amount of calcium in the diet after the lactation period (*p* < 0.05) and the magnitude of this decrease was significantly greater in lactating animals than in non-lactating animals, but in this case only those fed a diet containing 0.3% calcium (*p* < 0.05). Similar results were seen on the control side in animals fed diets containing 0.3% and 0.02% calcium (*p* < 0.05); however, the magnitude of this decrease was much greater in the lactating group [30]. In addition, the authors supplied histologic examination data revealing more details about ABL. The magnitude of dietary calcium-dependent decreases in interdental bone mass and the height of the alveolar bone crest (ABC) seemed to be greater in the lactating group. In more detail, height of the interdental ABC in the 0.3% calcium group, the distance between the ABC and cementum-enamel juntion (CEJ) of the first and second molars in lactating animals was significantly greater than in non-lactating animals, but in the control side there were differences among both groups (*p* < 0.01). On the other hand, although a similar decrease was seen in the 0.02% calcium group, the difference between the lactating and non-lactating groups was not statistically significant [30]. Animals were subject to a severe calcium deficiency for 20 days, followed by a recovery period of 20 days. Similar decrease in bone was observed in all sites during progression of calcium deficiency with a similar increase during recovery. Maximum bone loss increased to more than 50%. ABL was characterized by a reduction in trabecular bone without loss of alveolar crest height (ACH) [33]. In the remaining study, the effects of calcium-rich diet (by CaCO_3_ addition) against a regular diet were tested on mice inoculated with *Aggregatibacter actinomycetencomitans*. After 60 days, the number of osteoclasts was reduced in mice fed on diet rich in caclium, which led to diminished infection-induced alveolar bone loss. In addition, calcium-treated mice also presented decreased levels of tumor necrosis factor-α (TNF-α) in periodontal tissues (*p* < 0.05) [34].

### 2.2. Magnesium

Four studies, all reporting human observational surveys [22,36,37,38], were selected in relation to magnesium (Table 1). Magnesium was mainly measured in blood, although dietary intake was also estimated in one of them [40]. Moreover, in two of the selected articles, serum calcium/ magnesium ratio was considered due to a potential interaction between these two minerals’ effects. According to absolute serum levels, Freeland et al. [22] found no association with RPI. On the other hand, in another cross-sectional survey that considered people aged between 20 and 80 years old, a high serum calcium/magnesium ratio was associated with reduced PPD (*r* = −36.1, *p* < 0.001), less CAL (*r* = −42.8, *p* = 0.00) and a higher number of remaining teeth (*r* = 7.5, *p* = 0.019) [36]. Furthermore, the same study evaluated the effect of Mg-containing drug consumption in a subset of 180 individuals aged 40 years old and older. People taking magnesium-containing drugs showed less CAL (2.7 ± 1.6 vs. 3.5 ± 1.6, *p* < 0.01) and PPD (2.4 ± 0.6 vs. 2.8 ± 0.9, *p* < 0.01) compared with their matched counterparts (in a 1:2 matched-pair analysis for age, sex, smoking, and educational level). Magnesium intake was estimated in an investigation on hypertension risk factors and periodontitis relationship in (non-smokers and alcohol non-drinkers) women aged 46–58 years from Tanzania, but no correlations with the severity of periodontitis determined through CPITN score or number of teeth was observed [41]. No details about intake data collection were offered in the report, so its reliability is questionable. Finally, there was only a cohort study that reported an inverse dose-response relationship between calcium/magnesium ratio and periodontal disease events in elderly persons from the study in Niigata (Japan) citizens but only when smokers were considered, with ORs of 6.28 (95%CI: 1.45–27.28, *p* = 0.014) and 5.96 (95%CI: 1.30–27.34, *p* = 0.022), in first and second quartiles respect than the fourth, respectively [37].

### 2.3. Phosphorus

Three studies have been selected in humans concerning phosphorus [22,27,28], a cross-sectional study [22] and two case-control studies [27,28], which were already mentioned for the previous nutrients (Table 1). The cross-sectional study was the performed by Freeland et al. [22] who did not find correlations with levels in serum or diet. Concerning case-control studies, one was focused only on gingivitis in female adolescents from Rome [28] and different nutrient deficiencies (two thirds of recommended intake), but no association was found with deficient phosphorus intakes. In the remaining study that differentiated between aggressive and chronic periodontitis patients in a hospital from China, serum phosphorus levels were lower in both patient groups compared to healthy subjects (1.06–0.18 and 1.10–0.15 mmol/L, respectively vs. 1.26–0.17, *p* < 0.05) [27].

As for experimental studies in animals, three were found [41,42,43]. One of the reports was based on nutritional interventions in Syrian hamster (*Mesocricetus auratus*) using diets supplemented with different phosphate salts, so putative effects on findings that other minerals might have been involved should be considered. In these animals, a diet with 5% Ca_3_(PO_4_)_2_ led to inhibition of ABL index (43.3 vs. 45.9 in females, 45.8 vs. 50.9 in males). A similar effect was observed for a diet with a concentration of 1% (38.9 vs. 47.2 in females; 40.9 vs. 50.3 in males), while with a diet containing a Na_3_HPO_4_ and KH_2_PO_4_ mixture; the inhibition did not reach the level of significance [44]. It is very important to note the importance of the balance between calcium and phosphorus, from the point of view of dietary intake. The two remaining papers included a rat model of hyperparathyroidism in combination with experimentally induced periodontitis. Rats were fed a high calcium to phosphorus ratio (1:7) for 5 months until hyperparathyroidism symptoms appeared, and then, half of them received *E. coli* lypopolysaccharide (LPS) injections for one week to induce periodontitis. Rats with only dietary induced hyperparathyroidism (dHPT) demonstrated inflammatory and degenerative alterations in periodontium without pocket formation, but periodontitis was evident in groups that received endotoxin injections. Additionally, dHPT and periodontitis-induced rats revealed the highest amounts of gingival inflammatory cell and vessel counts, followed by the only periodontitis-induced rats and only dHPT group. The differences between groups were similar for CAL, bone losses, and osteoclast number [42]. In the other report, the same authors compared ABL and gingival levels of interleukin (IL)-1β and TNF-α between groups when rats were sacrificed. Hyperparathyroidism increased ABL associated to periodontitis (0.99 ± 0.07 vs. 0.70 ± 0.008 mm, *p* < 0.001), as well as gingival levels of IL-1β and TNF-α (92.25 ± 6.26 vs. 68.25 ± 5.23 pg/mL for IL-1β, *p* < 0.01; and 80.73 ± 4.52 vs. 62.85 ± 5.85 pg/mL for TNF-α, *p* < 0.05). Serum levels of proinflammatory cytokines was also measured before periodontitis induction and they were higher in rats with dHPT (15.75 ± 0.49 vs. 10.12 ± 0.92 pg/m, for IL-1β and 176.75 ± 23.06 vs. 13.02 ± 1.38 pg/mL for TNF-α, *p* < 0.001). Additionally they were positively correlated with the parathyroid hormone (PTH) levels in the experimental group (*r* = 0.93, *p* < 0.001 for IL-1β; and *r* = 0.94, *p* < 0.001 for TNF-α levels). At gingival level, differences were still present after two weeks, among rats without periodontitis (49.57 ± 4.50 vs. 26.05 ± 2.52 pg/mL for IL-1β, *p* < 0.01; and 34.39 ± 3.19 vs. 18.44 ± 1.87 pg/mL for TNF-α, *p* < 0.05), although the correlation with PTH levels presented 2-weeks before, was found just in non-injected rats (*r* = 0.93, *p* < 0.001 for IL-1β; and *r* = 0.79, *p* < 0.01 for TNF-α). Attending to these results, hyperparathyroidism induced by a high phosphorus diet may be responsible for the proinflamatory state that combined with LPS effect increased bone resorption [46].

### 2.4. Iron

Only one study was selected to clarify the relationship between dietary iron and periodontal disease [22]. Dietary intakes of iron in humans only were assessed in one cross-sectional study [22], but associations with RPI were not found. On the other hand, the selected study included information that would avoid estimating iron nutritional status but no relationship with RPI was found, as occurred with dietary intakes. Despite of these results, sideremia values alone has usually not been considered a good marker for iron levels in the body.

### 2.5. Copper

In humans, two cross-sectional studies [22,39] and a case-control study were selected [41] (Table 1). One of the cross-sectional studies chosen was the one performed by Freeland et al. [22] where the authors observed a direct and linearly relationship between serum copper level and RPI in single regression analysis (regression coefficient = 0.64, *p* < 0.001). Furthermore it was the second most significant factor in multiple regression analysis after age (which included other nutrient intakes or serum previously treated in this review) when an R^2^ improvement method was used. However, dietary intakes did not show any significant correlation with RPI (after Pearson correlation analysis) [22]. Despite these data, the association presented in this paper can be influenced by several reasons since no fasting instructions was given to patients before blood sample collection. In addition to that, dietary data may be not representative because of a dietary recall of only 24 h. The other was based on data from adult participants in the 2005 Japanese NHANES, where dietary intake of copper along with many other nutrients was assessed by a FFQ. Subjects were divided into two groups according to their individual maximum Community Periodontal Index (CPI) values (0–2 or 3–4) to subsequent analyses. After statistical univariate analysis, it was observed that copper intake was higher in subjects with higher CPI values (1.3 ± 0.4 vs. 1.3 ± 0.5, *p* = 0.026). However, no association was found in a logistic multiple regression analysis [35]. The case-control survey was also mentioned [41]. This includes health subjects and periodontitis patients systemically healthy and with DM2 from India, but differences were only was observed between systemically healthy and diabetes patients with periodontitis.

### 2.6. Zinc

Only five papers in relation to the effects of zinc on periodontal disease were found: two observational studies in humans [22,41] (Table 1) and three interventional trials (Table 2), one in humans [47] and two in rats [48,49]. Research in humans did not show any relevant role for zinc. Freeland et al. [22] have searched for associations among RPI and dietary intake or serum levels of zinc, but they did not find any. Serum zinc levels were also analyzed in the study in India that it has just been cited for copper [47], with the same results.

In a double-blind randomized-controlled trial (RCT) in children, zinc-supplemented syrup seemed to exert beneficial effects. Despite plaque index (PI) scores being similar at baseline, there were more children whose scores decreased in comparison to those that increased (18 vs. 6 children, *p* = 0.02), while those that receiving placebo syrup showed similar proportions. However, a decrease in Gingival Index (GI) occurred in more children in the two groups [47]. On the other hand, experiences in rats support a certain positive effect for dietary zinc. In one of the studies, a zinc-deficient diet was related to lower plaque (0.90 vs. 1.97, *p* < 0.001) and gingival indices scores (0.83 vs. 2.23, *p* < 0.001), but PPD were unaffected. Likewise, other oral histological abnormalities like ulcers (with a high rate of 29.9%) and hyperkeratosis, mainly in tongue, were more frequent in rats fed a zinc-deficient diet [48]. In the other study, the effects of zinc deficiency had similar consequences. Male Wistar rats fed a zinc-deficient diet showed higher GI than those fed zinc-containing diet (1.57 ± 0.76 vs. 0.50 ± 0.76; *p* = 0.001), and was the only that presented apthous ulcers in oral tissues [49]. However, as in the previous study, there was no significant difference regarding the PPD between two groups, nor regarding the epithelial and keratin thickening between two groups.

### 2.7. Potassium

Human research on dietary potassium role included in the present review was represented by two cross-sectional studies [22,38] and one case-control study [28], all mentioned above. The only result in support of a role of potassium comes from a cross-sectional study in (non-smokers and non-alcoholic) medium-age (46–58 years old) women from Tanzania [38]. In this group, a simple-regression analysis indicated that the severity of periodontitis was inversely correlated with 24-h urinary excretion of potassium (*r* = −0.579, *p* = 0.0004), that confirmed a link between potassium intake and food intake. However the rest of the papers did attribute any role to potassium. Freeland et al. [22] also assessed intakes, but did not find a significant association. In the case-control study based on females from Rome, no association in multivariate models was noted, although differences between means existed in the bivariate analysis [28].

### 2.8. Manganese

In relation to the possible role of manganese in periodontal disease, a cross-sectional study evaluating the association between plasma levels of manganese and periodontal status in a representative sample (*n* = 1679) of Korean adults (participants in the 4th Korean National Health and Nutrition Examination Survey) was published. Periodontal health was assessed by CPI and plasma levels were divided into quartiles to analyze its manganese association in a multivariate logistic regression analyses were performed (adjusted for sociodemographic variables, oral and general health behavior, oral health status, and systemic conditions). Like similar cross-sectional studies, this followed a stratified design and analyses were also performed in subgroups established according to variables whose interaction with periodontal status is known (namely, gender and smoking status). After analyses, some inverse relationships were observed between both variables with first-quartile (<1.057 mg/dL) showing a moderate association with CPI higher than 3, but only in males (OR = 2.13; 95%CI: 1.25–3.63) and current smokers (OR = 2.07; 95%CI: 1.04–4.11), compared to the fourth quartile (>1.544 mg/dL) [40]. Importantly, despite the fact the authors stated that these associations are significant neither a level of significance nor a confidence level were provided in the article.

### 2.9. Selenium

According to the establish selection criteria only a very recent observational report in humans was selected [42]. This was a case-control study on a sample of 150 non-smokers patients aged 30–60 years old from India, where the authors aimed to estimate the levels of glutathione, catalase, and Se in the serum of diabetic patients with periodontitis and healthy individuals with and without chronic periodontitis. When ANOVA and Tukey Honestly Significant Difference (HSD) multiple comparison test were carried out, they noted that Se levels were lower in diabetic patients with periodontitis and also in healthy individuals with periodontitis, but highest in healthy controls, showing the following values: 81.41 ± 55.419, 161.44 ± 84.787 and 193.84 ± 66.713, respectively (*p* ≤ 0.005), so it was concluded that serum levels of Se were inversely proportional to inflammation and tissue destruction.

## 3. Discussion

Findings from the studies collected in this review, overall, have a complex interpretation since the amount of minerals dosed and the variability in multiple features (sample, statistical analysis, markers used, experimental design, etc.). This makes it difficult to draw conclusions about the role of the different minerals in periodontal disease, particularly for those less studied. In most cases there is little evidence supporting a certain grade of relationship between a concrete mineral intake or nutritional status and periodontal health. There are many reasons that could explain this lack of evidence. Among them, the fact of the most of articles selected reported descriptive research. In addition to that, probably as a consequence of the methodological approach of this review, few articles studied in any depth the mechanisms responsible for the observed relationships.

However, there are other reasons inherent to studies investigating diet or nutrition role in a pathology or disease outcomes [50]. Nutrient excess or deficiencies usually lead to multifactorial pathologies or physiological states, so often we have only information of intakes for evaluation in observational studies. Further, possible relationships reported between nutritional status and disease outcomes are usually non-linear and typically exhibit a threshold effect [51]. This reinforces the utility of group subjects (percentiles or cut-off points) for statistical analysis as was done in most of the cases. Moreover, there is a much lower chance of finding significant differences in diseases if all groups are adequately nourished [50], as probably occurred in most of the populations studied. On the other hand, the methodology chosen to obtain data about nutrients also leads to other problems. Dietary intake estimates have been obtained for different methods including 24 h recalls, food diaries (between 3 to 7 days) and FFQs. However, 24-h recall and food diaries only represents current diet and obtaining accurate information with any of these three methods have certainly always have a degree of difficulty since the reporting of food intake by study participants is susceptible to memory errors and bias [50]. In support of these, there are also methods of determining nutritional status, such as measuring serum or plasma levels that alleviate the issues surrounding self-reporting of dietary intake (i.e., poor compliance). However, not all nutrients have meaningful biomarkers [52]. There are different reasons for that. For many nutrients, intake and biological availability are not necessarily equivalent, since age, medical conditions, meal composition and many other factors (genetics, smoking…) can affect their absorption and utilization by the body. Likewise, there are different factors (such as intestinal absorption, nutrient-nutrient and nutrient-medication interactions, certain diseases, or smoking) that might influence the amount of a nutrient actually available for tissues [50]. This is particularly important in the case of minerals since hormones like vitamin D_3_ and PTH have a key role in the absorption and excretion processes of some minerals and their levels and activity also depend on dietary intakes of various nutrients. In addition, circulating levels can reflect only intense and/or lasting dietary deficiencies, particularly for those minerals with great stores in mineralized tissues (i.e., calcium, phosphorus and magnesium).

Moreover, there was a wide variability with respect to evaluation of periodontal health status. In most of observational studies diagnosis of periodontal disease implied periodontal probing as part of the oral examination that allows obtaining CAL, PPD and BOP. However, studies used different periodontal disease definitions. On the one hand, such definitions could be based in PPD, CAL, or both combined and including or not the presence of BOP. In addition, conditions could have present just in one or a few sites or have to be generalized (30% of sites according to AAP) to consider that disease was present. However, currently it is assumed that PPD alone does not reliably reflect whether teeth have progressive CAL, except in sites with deep PPD. Anyway, in most of cases, CAL was used. Moreover, swelling of the gingival tissue due to inflammation may increase the depth of the pocket in cases where no loss of attachment has taken place. On the other hand, values chosen as cut-off point for diseases categories also were different. In other occasions, periodontal health was established using indexes. CPI or CPTIN (its former name) was the most frequent [40,44,45,50] and a value of 3 or more was chosen to separate individuals with periodontitis from healthy ones. However, because this index is based only on probing (PPD), a score of 3 or 4 denotes the presence of periodontal pockets, but provides no information on the presence or absence of BOP or calculus. In addition, RPI that are based on gingival inflammation and loss of periodontal attachment were also used but only in one study [22], although almost all minerals at both the serum and diet levels were analyzed in it. To complete this information two other indexes, both by Silness and Loe [53], have been used, although only in some experimental studies. These were PI that measures thickness of plaque on the gingival one third and GI that indicates the severity of inflammation. Finally, PPD, CAL and BOP also were used as disease outcomes alone to evaluate possible relationships with the severity of the disease, in different ways, mean values or percentage of sites with a minimum value.

Among the evaluated minerals, calcium is widely represented in the publications selected for the present review. There were much less information for the rest of minerals and in many cases and analyses they usually correspond to surveys that had other issues as their main objective. This mineral performs a key structural function as a component of the hydroxyapatite crystals of teeth and bone. The effect dietary calcium modifications has been clearly shown in systemic bone, with reductions in the rates of bone loss and osteoporotic fracture incidence in older persons as a result of providing calcium intake levels of at least 1000 mg daily in combination with vitamin D [54,55]. Because of its function and the effects of at systemic level, it was suggested many years ago that adequate calcium intake reduces the risks of oral bone loss and periodontal disease. In the present review, there are evidences for preventive effect of high dietary intakes on risk of periodontitis [20,36] or gingivitis [28] from some cross-sectional [20,21,36] and case-control studies [28]. Likewise, serum levels were inversely associated with risk of periodontitis progression [29]. However, there are others that showed no associations with intake [21,22,24]. In addition, studies reporting association were only carried out in certain population groups (pregnant women) or only showed associations in some age (younger) and/or sex (female) groups when stratified analyses were done. Anyway, differences among these results could be due to the different severity of the periodontitis considered or use of an index that could lead to underestimation of possible relationships. Moreover, many of them use linear regression analysis that could not detect associations when these are not linear. Associations and [20] lack of associations [22,27] were also reported in relation to serum levels in transversal studies. This could be due to subjects did not receive fasting instructions before blood extraction in the first case [20] although serum levels were adjusted for daily intake of calcium. Moreover, calcium levels are under negative feedback loop regulation by calcitonin and PTH, and associations with disease are more likely to be detected at extreme levels. Furthermore, the effect of dietary calcium amount may be dependent on the adequacy of other nutrients such as vitamin D and proteins [55]. In this respect, results from a study supporting this association, with an interesting approach suggest that calcium source could be a key factor since an inverse association of periodontitis risk only with intakes of total dairy calcium, from milk and fermented foods was found [25], in this case using a whole-population representative sample. Still, in spite of the fact FFQs were used in some surveys, data could represent current intakes in all cases, so causal relationships are unsure. For this results of longitudinal studies are very important, but in this case, an association has been only found for serum levels in one focused on elderly subjects. Interestingly, the correlation between Ca and periodontal disease has been also recently confirmed in a subset of dogs classified according to disease severity (from absent to severe periodontitis) [56]. In this case, researchers were only focused in inorganic Ca but it is expected that this calcium fraction was the most modifiable fraction by diet under normal conditions. Experimental studies in rodents have been carried out especially in physiological situations with high requirements of Ca, namely in pregnancy [31,33] and lactation [30], but in all cases dietary Ca amount positively influenced alveolar bone. Anyway, the effects of a Ca-rich diet on the periodontium was tested in a periodontitis mice model induced by bacterial inoculation directly affecting bone metabolism, including ABL and osteoclast number, it also led to a lower of TNF-α [34], which also suggests an interesting role in inflammation regulation for this mineral in this tissue. The role of dietary phosphorus or magnesium has also been investigated but this only seems relevant when their amounts are very different respect than calcium. Phosphorus is a component of DNA and ATP playing a key role in the energetic metabolism of cells. Moreover, it also form part of hydroxyapatite crystals together with calcium, which probably is the most interesting feature of this mineral in relation to periodontal disease since there are an important interaction between calcium and phosphorus metabolism. In support of this role, it has been reported that deficiencies led to dysfunction of organs and tissues with an important energy expenditure such as the nervous system and muscle whereas a great excess could lead to mineralization in some organs. Regarding studies included in this review, no clear evidence in humans for associations of phosphorus with periodontal disease were noticed [25,28]. This is expected since phosphorus deficiencies are rare in many populations and it usually are not primary (i.e., it does not depend on dietary intake). Additionally, serum levels that were measured in the two studies [25,28] are not good markers of intake. As with calcium, most of the body´s phosphorus is in bones and changes in dietary phosphorus do not necessarily imply variations in circulating levels. Actually, increased phosphorus levels could be a secondary consequence of calcium deficiency due to PTH-stimulated osseous resorption. Moreover, high PTH levels increase phosphorus excretion and decrease intestinal absorption. The importance of this interaction has been pointed out by studies showing an increased susceptibility to periodontal disease in animal with hyperparathyroidism induced by diets with unbalanced amount of phosphorus respect to calcium [45,46]. According to these studies, hyperparathyroidism induced by a high-phosphorus increased bone resorption but also may increase sensitivity to LPS-stimulation.

In contrast, results found in relation to magnesium seem more interesting. Magnesium is an essential cofactor in systems implicated in the synthesis and activation of nutrients. In addition, it is the second most prominent intracellular cation (after potassium) playing important roles in stabilization of membranes and ion transport. In addition, this element has important features linking it with calcium and phosphorus. Magnesium also influences bone and calcium metabolism due to the fact it stimulates calcitonin production (that acts as a preserving bone) and regulates PTH in a number of ways. Moreover, magnesium is found mainly (approximately two-thirds of the body´s total amount) in mineralized tissues that act as a storage reservoir, transferring magnesium into the blood stream when it is needed. Interestingly, an enhanced inflammatory response to bacterial product associated with reduced serum magnesium has also been reported. According to the publications collected in this review, there are few results supporting some effect for total daily magnesium intake on periodontal health. The only evidence was provided by a cross-sectional study where magnesium-containing drugs takers presented better periodontal health compared to non-takers [36]. On the other hand, when calcium/magnesium ratio has been taken into account, low values showed associations with periodontal disease in two investigations [22,40]. Anyway, only a cohort study was reported with respect to this concern, which was based on elderly people and only found high risk of suffering periodontal disease events in smokers [37]. Thus, to clarify this, at least more cohort studies in a sample representative of all population ages are needed.

**Figure 2:**
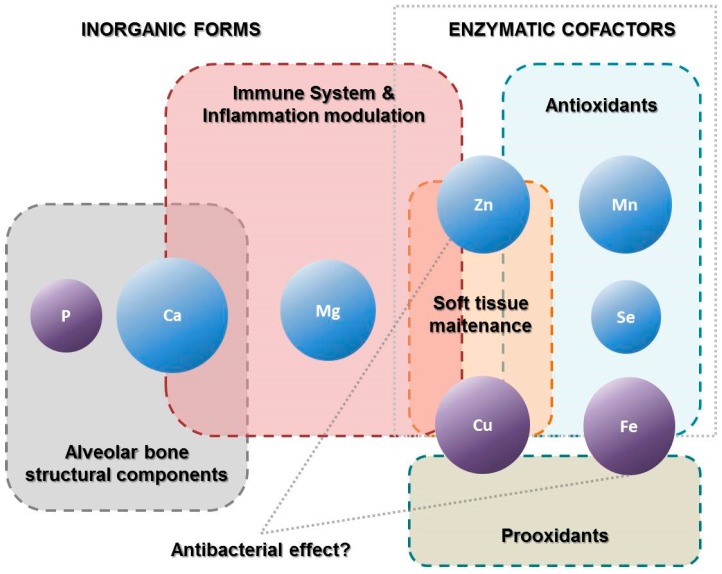
Role of minerals with possible implications in periodontal disease pathogenesis.

Another much evaluated mineral has been iron which is known for its function in oxygen transport as a component of the heme group. However, this is also a cofactor of several enzymes in the body and the electron transport chain. These include catalase and peroxidase, so iron has also an important antioxidant role to prevent tissue damage as a consequence of inflammation (Figure 2). On the other hand, excess of iron in the tissues results in the formation of free radicals and can lead to organ damage [57]. Thus, iron is essential in the diet but an optimal iron balance must be maintained. However, from the studies described here is not possible confirm any relationship, dietary intakes have been little evaluated and in most of case the only data available was serum levels of iron. Absence of differences in studies could be due to the fact the subjects in general were well-nourished. In addition, chosen biomarkers may be responsible since to adequately measure nutritional status all iron-binding compounds such as ferritin, haemin, lactoferrin and transferrin should be considered [58]. The importance of iron in bacterial growth and biofilm formation also has been suggested by some studies [59,60]. This could be an alternative mechanism for the implication of iron in periodontal disease.

Several studies have also evaluated possible effects of zinc and copper on periodontal health. Deficient nutritional status for any of these elements usually has a pleiotropic effect since they are components or at least are required in the medium for a multitude of enzymes [60,61]. Among systems involving these minerals, are included the immune system [9,22,61,62] and connective tissues [9,22,63] that has been shown to be affected by these minerals’ deficiencies which could have consequences on periodontal health. In addition, both minerals are found in cytosolic superoxide dismutase (SOD) [1]. In turn, copper can act as a pro-oxidant promoting tissue damage [64,65]. For this reason, it is necessary to maintain the proper dietary balance of copper along with other minerals [66]. Among the studies found, an inverse association with serum levels of copper was in a little cross-sectional study. However other two observational studies, among them one carried out in a sample of 3043 subjects, reported no association. In spite of the contradictory results that have been reported by observational studies in relation to zinc, research in rats confer importance to maintaining adequate levels of this mineral in the diet to prevent excessive inflammation in the periodontium [48,49]. However studies in humans, namely in children, have indicated that supplements are not effective in inflammation [47]. Curiously, a higher amount of dietary zinc led to lower PI scores in all cases [47,48,49]. This is in consistent with the negative effect on some bacterial colonies described for both cations [67,68,69,70]. Therefore, is possible that with zinc-deficient diets individuals would be more susceptible of suffer uncontrolled inflammation. Further, it is a possible that zinc had an additional antibacterial effect, but increasing dietary zinc over needs could be not important for periodontal disease onset.

Lastly, two additional trace elements have been evaluated in relation to periodontal health although research on this role is almost absent. In any case the results pointed out this potential relationship. These are selenium and manganese, two minerals that act cofactors of antioxidant enzymes, namely mitochondrial SOD in the manganese case and glutathione peroxidase in the case of selenium. Therefore they are considered as antioxidants due to the fact they help to minimize oxidative damage to lipid membranes. Although there are observational studies evaluating levels of these minerals in blood, inverse associations have been reported for manganese and selenium in a cross-sectional [42] and a case-control study [40], respectively.

## 4. Materials and Methods

### 4.1. Selection Criteria

Inclusion and exclusion criteria for the selection of papers for review were established prior to beginning the search in the online published literature. All relevant studies, both in humans and animals, investigating the association between periodontal disease and nutrient dietary intake or nutritional status markers of minerals were included, even when they included only people belonging to certain age groups or subject to special physiological conditions or illnesses (pregnancy, menopause, diabetes mellitus and others). Studies had to assess periodontal health condition, including also number or loss of teeth. However, it must be considered that the latter could be due to other pathologies. Regarding study design, cross-sectional, cohort and case-control studies were selected within those belonging to an observational type. On the other hand, only RCT were accepted from experimental surveys collected. Studies without design definition or enough details about it were removed. Additionally, interventional trials with nutrient combinations were excluded for individual assessment of each nutrient.

### 4.2. Information Source and Search Terms

MEDLINE: PubMed, the electronic database of the National Library of Medicine (Washington, DC, USA) was used to identify appropriate papers. We first derived two topics that were then combined by using the Boolean operator “AND”. Each theme was created by using the operator ‘‘OR’’ to combining different search terms. These appeared either as explorer text words or medical subject headings (Mesh) (in cases where they existed and were not contained within others already used). Periodontal disease-related terms were: periodontal, periodontitis, gingivitis and the following outcome measurements related to periodontal disease were also included: “Alveolar Bone Loss”[Mesh] OR “Periodontal Diseases”[Mesh] OR “Periodontal Attachment Loss”[Mesh] OR “Periodontal Index”[Mesh] OR “Gingival Hemorrhage”[Mesh] OR “periodontal disease” OR periodontitis OR “alveolar bone loss” OR “alveolar bone resorption” OR “tooth attachment” OR “tooth mobility” OR “gingivitis” OR “clinical attachment level” OR “periodontal attachment level” OR “attachment loss” OR “periodontal pocket” OR “pocket depth” OR “probing depth” OR “bleeding on probing” OR “gingival bleeding” OR “Gingival Hemorrhage” OR “gingival index” OR “bleeding index” OR “periodontal index”. In addition to this, a second topic related to nutrition or diet was created, where the search terms were the following: “Food”[Mesh] OR “Diet”[Mesh] OR “Eating”[Mesh] OR “Nutrition Surveys”[Mesh] OR “Nutrition Assessment”[Mesh] OR “Nutrition Therapy”[Mesh] OR “Nutrition Processes”[Mesh] OR “Nutritional Status”[Mesh] OR nutrition* OR nutrition OR nutrient* OR nutrient OR food OR dietary OR diet* OR intake OR intakes OR Consumption* OR Consumption OR Ingestion OR Eating.

### 4.3. Search Strategy

The search strategy aimed to find online published studies in the English language from inception of the database until May 2016. A comprehensive literature search was run independently by two persons. First, papers were screened by title and abstract. Screening procedures were adjusted for higher sensitivity (with restrictive search items omitted). Secondly, full text-papers were retrieved and selected based on the eligibility criteria, and duplicated studies were excluded. Titles without an abstract of which the title suggested that they were related to the objectives of this review were selected to screen the full text. Review authors’ disagreements or inconsistencies concerning inclusion of publications or extraction of data were discussed to eventually achieve mutual consensus.

### 4.4. Data Collection Process, Data Items and Summary Measures

The quantitative data extracted from papers included specific details about the interventions, populations, study methods, and outcomes of significance to the review question and specific objectives. Data from eligible studies were independently evaluated. When they were available, mean, differences among groups, and its standard deviations were included in results description. In the same sense, it was also collected association measurements (i.e., correlation or regression coefficients, IRRs, ORs, and their corresponding 95%CI), as well as significance levels or *p*-values considered, but only if significant associations and/or differences were found.

### 4.5. Quality Assessment and Risk of Bias

Subsequently, selected publications were fully-read to determine their quality, mainly through the assessment of risk of bias. The methodological quality of a publication was determined in accordance with several criteria depending on the study design. In the case of observational studies, the use of randomization (only in clinical trials), matching or restriction at the design level was taken into account, whereas the use of multivariate analysis, stratification or frequency matching was appraised at a data analysis level. Concerning bias, four types have been proposed: selection, detection, attrition and reporting [71]; although other authors have simplified this classification into two categories: selection and information [72]. Anyway, critical issues for preventing them depend on study design. In cross-sectional studies, three issues were mainly evaluated: sample randomization; reliability and objectivity of variable assessment, as well as definition of group if comparisons were made. In case-control studies, objectivity and reliability of evaluations of subjects that in this case including categories definitions and exposures evaluation method too were also taken into account. Lastly, in cohort studies, exposure and status definition, treatment duration and reliability and objectivity of outcome assessment were considered. Likewise, differences in intensity of medical surveillance, loss of follow-up, missing data treatment, and differences related to the outcome or exposure of risk factors between those who drop out and those who stay in the study were also evaluated. Inclusion criteria and/or sources of data or individuals background were considered for the establishing of the sample external validity and set of population represented. Additionally, grouping criteria in data analysis were taken into account when they were presented, especially if they were selected post hoc from alternative options.

On the other hand, intervention trials were assessed according to Cochrane guidelines, which take into account five type of bias: selection, performance, detection, attrition and reporting bias [73]. Based in its recommendations, the following domains were evaluated: sequence generation (randomization), allocation concealment, blinding of participants, operators and/or examiners, incomplete outcome data and selective reporting. In function of this assessment results, degree of risk (high, low or unclear) was established for each type of bias, as well as its possible magnitude and direction. The risk of bias and its possible effects were summarized for each outcome within each study. Finally, it was summarized for the review as a whole, where it was possible.

## 5. Conclusions

The heterogeneity of data and studies, as well as the lack of research on the question of this review for certain minerals, in general, lead to the conclusion that there is limited evidence to confirm a possible effect of dietary intakes for most minerals on the risk or severity of periodontal disease. For this reason more observational surveys with great sample sizes from representative groups for different minerals are needed. Additionally, consensus in periodontitis diagnosis is needed to improve our overall understanding of this subject. In any case, ensuring that healthy subjects or patients do not suffer from a specific nutritional deficiency is recommendable. Among all the minerals, calcium dietary intake seems important to maintain alveolar bone at least with intakes under recommendations in certain groups of risk or populations. Likewise, maintaining adequate proportions in the diet with respect to calcium of minerals that may influence bone and calcium metabolism (i.e., magnesium and phosphorus) also can be relevant. Moreover, some observations suggest that all those minerals with roles in immune systems (magnesium, zinc or copper) and/or antioxidant systems (magnesium, zinc, copper, manganese or selenium) should be considered in future research. Although there is no evidence to indicate that indiscriminately increasing the amounts ingested for the indicated minerals will reduce or help treat periodontal disease, it seems reasonable to suggest a mineral supplement when patients show inadequate nutritional status.

## Figures and Tables

**Table 1:** Observational studies on minerals’ associations with periodontal disease.

Study Type	Sample	Sex, Age, N	Dietary Intake Assessment	Nutritional Status Assessment	Periodontal Status	Association Assessment	Main Results/Conclusions	Ref.
CS	NHANES III participants (USA)	Both, ≥ 20 years, N = 11787	Dietary intake of Ca (<500, 500–799, & ≥ 800 mg/day) by 24-h recall	Serum levels ^1^ of total Ca	Periodontal disease (CAL > 1.5 mm)	Adj OR (*multiple logistic regression*)	Inverse association with intake & serum levels but only in certain age groups & gender	[20]
CS	2007–2008 DANHES participants (Denmark)	Both, ≥18 years, N = 3287	Dietary intake of Ca (*continous*, & *< & ≥ RDA*) by FFQ	-	Severe chronic periodontitis ^2^ mean CAL	Adj OR (*multiple logistic regression*) Adj β (multiple linear regression)	Negative association with mean CAL for intakes ≥ RDA	[21]
CS	Dental clinic patients (USA)	Both, N/A, N = 80	Dietary intakes of Ca, P, Fe, Zn & K by 24-h recall (N = 56)	Serum levels of Ca, Mg, P (Fe, Cu, Zn & K)	RPI	Linear correlation coefficient for intake Adj β for serum levels (*multiple linear regression*)	Inverse association with Cu serum levels	[22]
CS	Pregnant women (Japan)	Female, 31.5 ± 4 years, N = 1162	Dietary intake of Ca (*quartiles*) by DHQ	-	Periodontal disease (PPD ≥ 4 mm in ≥1 tooth)	Adj OR (*multiple logistic regression*)	Inverse association (*highest qt* vs. *lowest qt*)	[23]
CS	Non-smoker adults with ≥20 teeth (Japan)	Both, ≥18 years, N = 497	Dietary intake of Ca by 24-h recall	-	CPI & %BOP	Adj β (*multiple linear regression*)	No associations	[24]
CS	SHIP participants (Germany)	Both, 20–80 years, N = 4290	Ca-antagonists regular use & Mg-containing drugs intake	Serum Mg/Ca (quartiles)	% PPD ≥ 4 mm, %CAL > 4 mm, number of teeth	Adj OR (multiple logistic regression)	Inverse association of serum Mg/Ca with %PPD > 4 mm & %CAL > 4 mm, only in subjects aged ≥ 40 years (*lowest quartil* vs. *highest quartil*). People taking Mg-containing drugs showed less CAL	[36]
S	135 subjects from 3rd follow-up of COHSS (Denmark)	Both, >65 years, N = 135	Dietary intakes of dairy food & Ca (100 g increments) by DHI	-	Periodontitis (number of teeth with additional CAL ≥ 3 mm)	Adj IRR (*multiple logistic regression*)	Inverse association with intakes of total dairy Ca, from milk & fermented foods	[25]
CS	non-smokers & non-alcoholic women from CARDIAC study (Tanzania)	Female, 46–58 years, N = 81	Dietary intake of Mg (N/A) & 24-h urinary excretions of K	-	number of teeth, & CPITN	Simple correlation coefficients	Negative association of K urinary levels with CPITN	[38]
CS	NHANES non-pregnant participants (Japan)	Both, ≥20 years, N = 3043	Dietary intake of Cu by 24-h recall	-	CPI = 3–4	Adj OR (*multiple logistic regression*)	No association	[39]
CS	KNHANES participants (South korea)	Both, ≥19 years, N = 1679	-	Whole blood levels of Mn (quartiles)	CPI ≥ 3	Adj OR (*multiple logistic regression*)	Inverse asociation	[40]
CC	Female non-smoker adolescents (Italy)	Female, 17–19 years, N = 54	Dietary intake of Ca, P & K (<2/3 RDA) by 3-days record	-	Gingivitis-affected (≥1 site with BOP) vs. non-affected	Differences between groups	Negative association of Ca intake with gingivitis risk	[28]
CC	Subjects from a Health Center (China)	Both, 16–64 years, N = 178	-	Plasma level of Ca & P (quartiles)	Aggressive periodontitis vs. chronic periodontitis—affected ^3^ vs. healthy (Staff)	Differencies among groups (ANCOVA)	Patints had lower levels of P	[27]
CC	Non-smokers outpatients (India)	Both, 30–60 years, N = 60	-	Serum levels of Cu & Zn	DM2 & periodontal disease-affected vs. only periodontal disease-affected (30% sites with CAL ≥ 5 mm & BOP) vs. healthy	Differences among groups	Subjects with perioedontitis showed higher Zn levels respect to than those with DM2	[41]
CC	Non-smokers individuals with ≥20 teeth (India)	Both, 30–60 years, N = 150	-	Serum levels of Se	DM2 & chronic periodontitis-affected ^4^, only chronic periodontitis-affected ^4^ vs. healthy	Differences between groups	Subjects with periodontitis (with or without DM2) showed the lowest Se levels	[42]
C (5 years)	MONICA study participants (Denmark)	Both, 30–60 years, N = 2113	Dietary intakes of Ca (total, E-adjusted as well as < & ≥ RDA) by 7-days record	-	Tooth loss	Adj IRR (*multiple logistic regression*)	Inverse association only in men	[26]
C (6 years)	Niigata city citizens (Japan)	Both, 70 years, N = 266	-	Serum levels of Ca	Progression of periodontal diseae (number of teeth with additional CAL ≥ 3 mm)	Adj β (*multiple linear regression*)	Negative association	[29]
C (6 years)	Niigata city inhabitants (Japan)	Both, 73 years, N = 309	-	Serum Ca/Mg (quartile*s*)	Periodontal disease events (CAL ≥ 3 mm/year at any teeth)	Adj OR (*multiple logistic regression*)	Negative association of Ca/Mg ratio with periodontal disease events only among smokers	[37]

^1^ Serum levels were adjusted by daily intake of calcium in subsequent analyses, ^2^ ≥2 inter-proximal sites with CAL ≥ 6 mm in different teeth & ≥1 inter-proximal site with PPD ≥ 5 mm. ^3^ according to IWCPDC criteria [43]. ^4^ according to AAP/CDC criteria for severe generalized chronic periodontitis. Abbreviations: %: percentage of sites, β: linear regression coefficient, AAP: American Association of Periodontology, Adj: adjusted, ABL: alveolar bone loss, AL: attachment loss, ANCOVA: Analysis of covariance, BMD: bone mass density, BOP: bleeding on probing, C: cohort study, Ca: calcium, CAL: clinical attachment loss, CARDIAC: Cardiovascular Diseases and Alimentary Comparison, CC: case-control study, CDC: Center for Diseases control and Prevention, COHSS: Copenhagen Oral Health Senior Study, CPI: Community Periodontal Index, CPITN: Community Periodontal Index Treatment Needed, CS: cross-sectional study, Cu: Copper, DHI: diet history interview, DHQ: diet history questionnaire, DM2: type 2 diabetes mellitus, Fe: iron, FFQ: food frequency questionnaire , GI: Gingival Index, h: hours, IRR : incidence rate ratio, IWCPDC: International Workshop for the Classification of Periodontal Diseases and Conditions in 1999 [39], K: potassium, KNHANES: 4th Korean National Health and Nutrition Examination Survey, v m: months, Mg: magnesium, Mn: Manganese, MONICA: Monitoring Trends and Determinants in Cardiovascular Disease, N: sample size, N/A: not available, NHANES: National health and Nutrition Examination Survey, NHANES III: Third National Health and Nutrition Examination Survey, OR: *odds* ratio, P: phosphorus, PI: Plaque Index, PPD: periodontal probing depth, RDA: recommended daily amount, RPI: Russel´s Periodontal Index, Se: selenium, SHIP: Study of Health in Pomerania; SOF: Study of Osteoporotic Fractures, USA: United States of America, vs: versus, Zn: Zinc.

**Table 2:** Experimental studies on minerals effects on periodontal disease.

Subjects/Animals, Age, Sample Size (N)	Experimental Design (Duration)	Main Results/Conclusions	Ref.
Pregnant & non-pregnant Wistar rats, 10 weeks, N = 62	Diets containing 0.9%, 0.3%, or 0.02% Ca (6 weeks). In pregnant rats, it includes gestation & lactation periods (3 weeks each)	BMD of alveolar bone decreased with Ca intake, but this decrease was greater in pregnant rats. Mother’s intake also affected to Pups‘ BMD	[31]
Pregnant (lactating) & non-pregnant (non-lactating) Wistar rats, 10 weeks, N = 62	Diets containing 0.9%, 0.3%, or 0.02% Ca (46 days) with experimental periodontitis induced by an elastic ring (last 2 weeks). Pregnant rats started lactation on d 25	BMD & ACH decreased in periodontitis side according to the Ca intake, but this was greater in the lactating group	[30]
Male BALB/c mice inoculated with *Aact*, 18 weeks, N = 40	Regular or CaCO_3_-rich diet (30 or 60 days)	Ca-rich diet fed animals showed lower osteoclasts, ABL & levels of TNF-α in periodontal tissues	[34]
Syrian hamsters, N/A, N = 10	Diet with 5% Ca_3_(PO_4_)_2_ or a mixture of Na_2_HPO_4_ & KH_2_PO_4_ (90 days or 150 days)	Ca_3_(PO_4_)_2_-rich diet inhibited ABL	[44]
Male SpDw rats, 3 months, N = 24	Ca/P imbalanced diet to induced dHPT or standard diet (5 months), following by LPS- or saline-injections (next 2 weeks)	Rats with dHPT & periodontitis revealed the highest amounts of inflammatory cells & vessels as well as of ABL & AL, followed by rats with only periodontitis & with only dHPT.	[45]
SpDw rats, Adults, N = 24	Ca/P imbalanced diet to induced dHPT or standard diet (5 months), following by LPS- or saline-injections (next 2 weeks)	IL-1β & TNF-α were highest in rats with dHPT & periodontis, followed by those with only LPS-treated. They were positively correlated to PTH levels in rats with only dHPT	[46]
Children (India), 8.50 ± 0.7 years, N = 68	Orally-administed elemental Zn supplements or placebo syrup ^DB^ (10 weeks)	Zn supplements improved PI, but not GI	[47]
SpDw rats, at weaning, N = 14	Zn-deficient or normal diet (4 weeks)	Rats fed Zn-deficient diet showed higher PI & GI. No differences were observed for PPD score	[48]
male Wistar rats, at weaning (24 days), N = 14	Zn-deficient or Zn-containing diet (4 weeks)	Aphthous ulcer on the floor of the mouth was observed in Zn-deficient rats, which showed higher GI. No differences were observed for PPD score	[49]

Abbreviations: ABL: alveolar bone loss, ACH: alveolar bone crest height, *Aact: Agregatibacter actinomycetencomitans*, AL: attachment loss, BMD: bone mass density, Ca: calcium, DB: double blind, dHPT: dietary induced hyperparathyroidism, GI: Gingival Index, IL: Interleukin, IV: in vivo study, K: potassium, LPS: lipopolysaccharide; Na: sodium, P: phosphorus, PI: Plaque Index, PPD: periodontal probing depth, PTH: Parathyroid Hormone, SpDw: Spargue-Dawley, SW: Swiss-Webster, TNF-α: Tumor necrosis factor alpha, USA: United States of America, Zn: Zinc.
